# The Usefulness of the TOAST Classification and Prognostic Significance of Pyramidal Symptoms During the Acute Phase of Cerebellar Ischemic Stroke

**DOI:** 10.1007/s12311-015-0676-6

**Published:** 2015-06-04

**Authors:** Edyta Dziadkowiak, Justyna Chojdak-Łukasiewicz, Maciej Guziński, Leszek Noga, Bogusław Paradowski

**Affiliations:** Department of Neurology, Wroclaw Medical University, ul. Borowska 213, 50-556 Wroclaw, Poland; Department of Neuroradiology, Wroclaw Medical University, ul. Borowska 213, 50-566 Wroclaw, Poland; Department of Pathophysiology, Wroclaw Medical University, ul. Borowska 213, 50-556 Wroclaw, Poland

**Keywords:** Cerebellar stroke, TOAST, Vascular territory of stroke, Pyramidal signs

## Abstract

Cerebellar stroke is a rare condition with very nonspecific clinical features. The symptoms in the acute phase could imitate acute peripheral vestibular disorders or a brainstem lesion. The aim of this study was to assess the usefulness of the Trial of Org 10172 in Acute Stroke Treatment (TOAST) classification in cerebellar stroke and the impact of clinical features on the prognosis. We retrospectively analyzed 107 patients with diagnosed ischemic cerebellar infarction. We studied the clinical features and compared them based on the location of the ischemic lesion and its distribution in the posterior interior cerebellar artery (PICA), superior cerebellar artery (SCA), and anterior inferior cerebellar artery (AICA) territories. According to the TOAST classification, stroke was more prevalent in atrial fibrillation (26/107) and when the lesion was in the PICA territory (39/107). Pyramidal signs occurred in 29/107 of patients and were more prevalent when the lesion was distributed in more than two vascular regions (*p* = 0.00640). Mortality was higher among patients with ischemic lesion caused by cardiac sources (*p* = 0.00094) and with pyramidal signs (*p* = 0.00640). The TOAST classification is less useful in assessing supratentorial ischemic infarcts. Cardioembolic etiology, location of the ischemic lesion, and pyramidal signs support a negative prognosis.

## Introduction

Ischemic cerebellar infarction is a rare condition and accounts for between 1.5 % and 20 % of all ischemic strokes. It is common between the fifth and eighth decades of life, with men aged 60 to 65 being affected more often than women [1–5]. The risk factors for cerebellar stroke are the same as for strokes in other areas of the brain. Clinical manifestations in the acute stage are very nonspecific, an isolated vertigo may mimic acute peripheral vestibular disorders or a brainstem lesion [3, 4, 6–10]. Also, brain computed tomography (CT) is less accurate in detecting an ischemic lesion in the first hours. Diffusion-weighted imaging (DWI) is highly sensitive in detecting ischemic changes in the subacute stage of stroke [11–13]. The aim of the study was to assess the presence of the pyramidal symptoms in the acute phase of cerebellar infarction, its impact on the prognosis, and the usefulness of the Trial of Org 10172 in Acute Stroke Treatment (TOAST) classification in specifying the etiology of cerebellar stroke.

## Material and Methods

We retrospectively analyzed medical history of adult patients with a diagnosis of cerebellar infarction hospitalized at the Department of Neurology, Medical University and at the Department of Neurology, Marciniak Hospital from 1 January 2008 to 31 December 2011. Diagnosis of stroke was defined according to the WHO criteria [14]. Cerebellar strokes were classified into five categories based on etiology, using the TOAST classification: 1) large-artery atherosclerosis, 2) cardioembolic stroke, 3) small-artery occlusion (lacunes), 4) stroke of other determined cause, and 5) stroke of undetermined cause. We also analyzed the symptoms in the acute phase of stroke and the presence of pyramidal symptoms. The affected vascular territories were specified using CT and/or brain MRI during hospitalization for acute and subacute stroke. All patients with stroke underwent neuroimaging studies including CT and MR examinations in acute and subacute stroke. The CT studies were performed using 64-row and 16-row scanners (GE Healthcare) with a slice thickness of 0.6 mm and included unenhanced and contrast-enhanced CT examinations. All MR studies were performed using a 1.5-T MR scanner (GE Healthcare). The analyzed MR studies included standard MR examination (T1-, T2-weighted images, FLAIR sequence, and DWI) without or with contrast administration.

In the cerebellum, there are three major arteries on each side: the superior cerebellar artery (SCA), the anterior inferior cerebellar artery (AICA), and the posterior interior cerebellar artery (PICA). Radiological images with artifacts were excluded from the analysis of the affected vascular territories.

The evaluation study was conducted with the participation of neurologists, a very experienced neuroradiologist, and a neurology resident.

Pearson chi-square tests or Fisher’s exact tests were used to compare frequencies. All statistical analyses were performed with a significance level of alpha = 0.05.

## Results

The demographic data, clinical characteristics, risk factors, and the affected vascular territories are shown in Table 1. The most common risk factor was hypertension, observed in 61 % of the patients. The PICA was the most frequently involved territory at 36 %, and the left hemisphere was most often affected. Hydrocephalus occurred in only 3/107 cases and was mainly associated with PICA infarcts. These patients were treated with external ventricular drainage. Table 2 summarizes clinical features according to the vascular territory of the ischemic lesion. The most common clinical symptoms were vertigo or dizziness (70/107) and gait imbalance. The gait disturbance was more prevalent in stroke in the PICA and SCA territories (*p* = 0.01292). In patients with lesions in more than one vascular territory, the pyramidal signs such as reflex asymmetry and Babinski signs were more common (*p* = 0.0009). Table 3 demonstrates etiology of cerebellar stroke according to the TOAST classification. Atrial fibrillation was the most prevalent risk factor of cerebellar stroke. The PICA stroke was caused by atherothrombosis verified by Doppler ultrasound examination. According to the TOAST classification, no identifiable etiology was present in 51 % of patients (55/107). Table 4 presents the percentage of deaths based on the affected vascular territory, the TOAST classification, and presence of pyramidal signs. Death was more probable among patients with ischemic focus caused by cardioembolism (*p* = 0.00094) with more than one vascular territory affected (*p* = 0.00185) and with the presence of pyramidal symptoms (*p* = 0.00640).

**Table 1:** Characteristics of patients with the cerebellar ischemic stroke including the affected vascular territories

Period of observation: 2008–2011				
The number of patients, F/M (Female/Male)	107, 36 (34 %) / 71 (66 %)			
The average age	64.6+/−12.06 (27–87)			
	F:64+/−14.22 (27–83)			
	M:66+/−10.89 (44–87)			
Risk factors				
Hypertension	Atrial fibrillation	Diabetes	Ischemic disease	Myocardial infarction
65 (61 %)	31 (29 %)	22 (21 %)	30 (28 %)	16 (15 %)
Vascularization territory (*n*/%)				
PICA	SCA	AICA	More than 1 vessel	
39 (36 %)	28 (26 %)	7 (7 %)	33 (31 %)	
Deaths (*n* = 20) and the extent of vascularization				
2	1	0	17

**Table 2:** Clinical symptoms depending on the territory of the cerebellar ischemic stroke

Vascularization territory	Symptoms							
	Systemic vertigo	Non-systemic vertigo	Imbalance disorders	Hearing disorders	Swallowing disorders	Hoarseness	Vomiting	Pyramidal symptoms
PICA (*n* = 9)	14	19	27*	6	4	3	20	5
SCA (*n* = 28)	9	10	20	3	1	3	8	7
AICA (*n* = 7)	1	3	4	0	1	0	4	1
More than 1 vessel (*n* = 33)	9	5	12	2	6	3	5	16 **
Total (*n* = 107)	33	37	63	11	12	9	37	29

*p = 0.01292, **p = 0.0009

**Table. 3:** TOAST distribution among the patient with the cerebellar ischemic stroke

TOAST	Number	Percent (%)
Large-artery atherosclerosis	26/107	24
Cardioembolism	26/107	24.3
Small-vessel occlusion	0/107	0
Stroke of other determined etiology	0/107	0
Stroke of undetermined etiology	55/107	51

**Table 4:** Analysis of the incidence of death depending on the vascularization territory, TOAST, and the presence of pyramidal symptoms in cerebellar ischemic stroke

Death	TOAST distribution			Vascularization territory				Pyramidal symptoms
	Large-artery thrombosis	Cardioembolism	Undetermined etiology	PICA	SCA	AICA	More than 1 vessel	
Yes (*n*/%)	1 (3.85)	11 (42.31)*	8 (14.55)	2 (5.13)	1 (3.57)	0 (0.0)	17 (51.52)**	11 (55.00)***
No (*n*/%)	25 (96.15)	15 (57.69)	47 (85.45)	37 (94.87)	27 (96.43)	7 (100.0)	16 (48.48)	9 (45.00)

*p = 0.00094, **p = 0.00185, ***p = 0.00640

## Discussion

Our study confirms data from the literature showing more frequent cerebellar ischemic stroke among men [1, 2, 15]. As in ischemic stroke, the primary factors of cerebellar infarction are hypertension and atrial fibrillation. Other risk factors are less common. Cardiac sources of cerebellar stroke increase the risk of mortality.

The location of ischemic lesion may suggest the etiology of cerebellar stroke. Most strokes in PICA territories arise from the atherosclerosis of the basilar artery, while the majority of strokes in the SCA range are associated with embolic mechanism. Ischemic focus in the AICA territory is often associated with a local thrombotic process or cardioembolism [15–23]. In our material, large-artery atherosclerosis was found significantly more often in the territory of PICA circulation infarct, much like in the territory of anterior circulation infarct. Atrial fibrillation occurred mostly among people with infarction distribution in SCA [6] people and PICA [5]. Similar findings were reported by Chung [17], who studied the relation between the mechanism and the vascular territory of a stroke lesion and showed that cardioembolism was most frequent in superior cerebellar artery territory infarction (60 %).

The usefulness of the TOAST classification was confirmed in supratentorial ischemic stroke to determine prognosis, prediction, and therapy in the acute phase and secondary prevention. Based on the assessment of our group of patients, it was shown that the TOAST classification is less useful in assessing supratentorial ischemic infarcts. The cause of stroke could not be clearly determined in 50 % of patients. The TOAST classification has various limitations related to, among other factors, genetics (for instance, two polymorphisms on chromosome 4q25, associated with atrial fibrillation, increased the risk of cardioembolic or cryptogenic stroke), race, ethnicity, and age. Jaffre et al. [24] showed the differences in the risk factor profile between etiological subtypes of ischemic stroke in the young. In this study, cardioembolism was correlated with age, atherothrombotic stroke was correlated with diabetes, hypertension, smoking, and age, while small-vessel disease was correlated with hypertension and age. Therefore, it is necessary to find new criteria to classify ischemic stroke subtypes, such as the ASCO classification (Atherothrombosis, Small-vessel disease, Cardiac causes, and Other common causes) or CCS classification (Causative Classification Stroke System) [24–29].

The cerebellum is supplied by the PICA, which arises from the vertebral artery and supplies the dorsolateral part of the medulla oblongata, the inferior surface of the cerebellum, and the choroid plexus of the fourth ventricle. In the case of ischemia covering the inferior cerebellar vermis and vestibulocerebellum, supplied by the ramus medialis, clinical symptoms consist of severe vertigo, ataxia, and nystagmus. In the case of ischemic hemispheres of the cerebellum supplied by the ramus lateralis, there may appear nausea, vomiting, vertigo, gait ataxia, and ataxic limbs with dysmetria, conjugate gaze palsy, dysarthria, and miosis. In an extensive infarction of this territory, disturbances of consciousness, hydrocephalus, or brain stem compression can be observed. PICA occlusion can also lead to Wallenberg syndrome [8–10, 30–36]. Among our patients, infarction in the PICA distribution was reported most frequently. The clinical picture was dominated by systemic and non-systemic vertigo and imbalance. Less frequently, there were hearing disorders. Isolated occurrences of systemic vertigo, suggesting misdiagnosis of vestibular neuronitis, were observed in 33 of our patients, including more than 14 with infarction in the PICA distribution.

The superior surface of the cerebellum is supplied by SCA, whose obstruction leads to damage of the superior cerebellar peduncles. The characteristic clinical syndromes in the territory of the SCA are ataxia/abasia and intention tremor. However, vertigoes are rarely observed in the clinical picture. Nystagmus is caused by a lesion of the medial longitudinal fasciculus and the dorsal spinocerebellar tract. Occurring homolaterally to the focus, the Horner syndrome is associated with damage to the descending sympathetic fibers [6, 37–39]. In the group of patients, the ischemic lesion with infarction in the SCA distribution was observed in 26 % of patients. The clinical picture was significantly dominated by imbalance and systemic and non-systemic vertigo. In the infarction group of the SCA range, 7 of our patients had pyramidal signs not connected with supratentorial focus. Pyramidal symptoms appeared significantly more often in areas covering more than one cerebellar vessel. Other symptoms were shown in Table 2.

The anterior part of the undersurface of the cerebellum and the inferior part of the pons are supplied by AICA arising from the basilar artery. Obstruction of the vestibular artery that supplies blood to the inner ear causes vertigo, nausea, vomiting, and nystagmus. Accession of the spinal trigeminal nucleus can cause facial hypesthesia to pain and temperature and diminished corneal reflex. Ischemic stroke, including bulb and lateral parts of pontine tegmentum, can cause paralysis of facial muscles and deafness. In the case of sympathetic fiber disturbance, Horner’s syndrome may occur on the damaged side. Damage of cerebellar and cerebral peduncles causes homolateral ataxia, and accession of lateral spinothalamic tract causes contralateral hypesthesia to pain and temperature in the limbs and trunk [40–48]. Among our patients, imbalance and vomiting dominated. Clinical symptoms in acute cerebellar stroke are nonspecific and may mimic acute vestibular syndrome. Also, negative CT in the acute phase does not facilitate cerebellar stroke diagnosis.

Death was mostly observed in patients with ischemic focus caused by cardiac sources in the case of the focal range of infarction in the PICA and SCA distributions and significantly more often among patients with pyramidal symptoms and the focal area involving more than one vessel of vascular territories of the cerebellum. Contrasting results were obtained in several research studies, which found high mortality in patients with stroke of undetermined etiology [49–51]. Based on the TOAST classification, D’Anna et al. [49] showed a higher risk of death or high disability (*p* < 0.0001) and a worse 6-month case fatality (*p* < 0.0001) among the patients with stroke of undetermined etiology. By contract, Paradowski and Maciejak [52] found no significant differences in TOAST classification in the patients with ischemic stroke, treated in neurological or internal medicine wards. We suggest that inconsistent findings regarding mortality in patients with stroke could be explained by the relatively younger ages of patients (on average 64.6 years old) and the high rate of cardioembolism in our study. Additionally, there was a dilution effect caused by the limitation present in TOAST classification.

In conclusion, our study showed that the TOAST classification does not uniquely determine the cause of cerebellar stroke in more than half of the patients. Some poor predictors of acute stroke in the cerebellum include the location of the focus, the etiology of embolism, and the presence of pyramidal symptoms deriving most often from ischemia of two territories of cerebellar vascularization.
